# Complex evolutionary history of photosynthesis in *Bradyrhizobium*

**DOI:** 10.1099/mgen.0.001105

**Published:** 2023-09-07

**Authors:** Juanita R. Avontuur, P. Markus Wilken, Marike Palmer, Martin P. A. Coetzee, Tomasz Stępkowski, Stephanus N. Venter, Emma T. Steenkamp

**Affiliations:** ^1^​ Department of Biochemistry, Genetics and Microbiology (BGM), Forestry and Agricultural Biotechnology Institute (FABI), University of Pretoria, Pretoria, South Africa; ^2^​ School of Life Sciences, University of Nevada Las Vegas, Las Vegas, NV, USA; ^3^​ Department of Biochemistry and Microbiology, Institute of Biology, Warsaw University of Life Sciences (SGGW), Warszawa, Poland

**Keywords:** *Bradyrhizobium*, comparative genomics, photosynthesis gene cluster, phylogenetics, synteny

## Abstract

*
Bradyrhizobium* comprises a diverse group of bacteria with various lifestyles. Although best known for their nodule-based nitrogen-fixation in symbiosis with legumes, a select group of bradyrhizobia are also capable of photosynthesis. This ability seems to be rare among rhizobia, and its origin and evolution in these bacteria remain a subject of substantial debate. Therefore, our aim here was to investigate the distribution and evolution of photosynthesis in *Bradyrhizobium* using comparative genomics and representative genomes from closely related taxa in the families *Nitrobacteraceae, Methylobacteriaceae, Boseaceae* and *Paracoccaceae*. We identified photosynthesis gene clusters (PGCs) in 25 genomes belonging to three different *Bradyrhizobium* lineages, notably the so-called Photosynthetic, *B. japonicum* and *B. elkanii* supergroups. Also, two different PGC architectures were observed. One of these, PGC1, was present in genomes from the Photosynthetic supergroup and in three genomes from a species in the *B. japonicum* supergroup. The second cluster, PGC2, was also present in some strains from the *B. japonicum* supergroup, as well as in those from the *B. elkanii* supergroup. PGC2 was largely syntenic to the cluster found in *Rhodopseudomonas palustris* and *Tardiphaga *. Bayesian ancestral state reconstruction unambiguously showed that the ancestor of *Bradyrhizobium* lacked a PGC and that it was acquired horizontally by various lineages. Maximum-likelihood phylogenetic analyses of individual photosynthesis genes also suggested multiple acquisitions through horizontal gene transfer, followed by vertical inheritance and gene losses within the different lineages. Overall, our findings add to the existing body of knowledge on *Bradyrhizobium *’s evolution and provide a meaningful basis from which to explore how these PGCs and the photosynthesis itself impact the physiology and ecology of these bacteria.

## Data Summary

The authors confirm all supporting data, code and protocols have been provided within the article or through supplementary data files. All supplementary material has been deposited and is available in the Microbiology Society’s data repository Figshare account: https://doi.org/10.6084/m9.figshare.24024765 [1].

**Impact Statement**

*
Bradyrhizobium* is well known for its nitrogen-fixation symbiosis with legumes, but some strains are also capable of photosynthesis. Previously, this trait was thought to be limited to a select group of strains in *Bradyrhizobium* (known as the Photosynthetic lineage), yet the ever-increasing genome sequence data indicate many more species might be capable of photosynthesis. Here, we investigated the distribution and evolution of the photosynthesis gene cluster across diverse bradyrhizobia using a combination of approaches involving comparative genomics, predictions of ancestral phenotypes and phylogenetics. Our results showed that several of these bacteria from disparate lineages encode one of two photosynthesis gene cluster architectures. Contrary to previous studies, our analyses also indicated that these gene clusters were probably absent from the ancestor of *Bradyrhizobium *. The extant distribution patterns observed are best explained by multiple horizontal acquisitions, followed by vertical inheritance, multiple gene replacements and losses. Our findings provide a foundation for exploring the evolution of photosynthesis genes in the genus *Bradyrhizobium* and the broader *Nitrobacteraceae *.

## Introduction

*Bradyrhizobium* forms part of the family *Nitrobacteraceae *, also known as the *Bradyrhizobiaceae* (order *Hyphomicrobiales *, class *Alphaproteobacteria *, phylum *Pseudomonadota *) [2]. *Nitrobacteraceae* currently includes 11 genera [3] of ecologically diverse bacteria [4]. The family consists of plant endophytes and symbionts [5, 6], autotrophs [6–10], nitrifiers [9] and various other soil bacteria [11–13], as well as animal pathogens [14]. Among these genera, *Bradyrhizobium* is the most widely studied due to its inclusion of species capable of nodulation-associated diazotrophy in symbiosis with legumes or plants in the genus *Parasponia* [4, 15]. These nodulating bacteria, collectively referred to as rhizobia, induce the formation of specialized plant organs (i.e. nodules) in which they fix atmospheric dinitrogen to ammonia or its derivatives. Their symbiotic ability is usually conferred by nodulation (*nod*) genes, the expression of which culminates in formation of Nod-factors for initiating and establishing the symbiosis with the legume [16].

Members of *Bradyrhizobium* are abundant in various soil and rhizosphere ecosystems, where they display diverse lifestyle traits [4, 17, 18]. In addition to nodule-based nitrogen-fixation in symbiosis with legumes, they can be endophytic by colonizing the roots of legumes and non-legume plants, free-living diazotrophs, and free living and not display any of these traits, or they can display various combinations of these traits [19–21]. There are also some strains capable of photosynthesis, a trait that is well-understood in certain other *Pseudomonadota* species, cyanobacteria and a number of other bacterial groups [22]. In *Bradyrhizobium *, photosynthesis is thought to be limited to the so-called ‘Photosynthetic supergroup’ [17, 23] whose members (e.g. *B. oligotrophicum* and *B. denitrificans *) are predominantly symbionts of species in the *Aeschynomene* genus of aquatic legumes [23–25].

The varied physiological and ecological properties of *Bradyrhizobium* species have promoted much debate on the taxonomic status of this genus. For example, the ability to photosynthesize was used as support for recognizing the Photosynthetic supergroup as a distinct and potentially separate genus [26]. However, such a split is not supported by phylogenomic data, which typically resolve *Bradyrhizobium* as monophyletic with the Photosynthetic supergroup nested within the overall phylogeny of the genus [17, 27]. Genome analysis also revealed that photosynthesis is probably not limited to the Photosynthetic supergroup, as various *Bradyrhizobium* species outside this lineage encode genes involved in the trait [17, 20, 28–30].

In the Photosynthetic supergroup of *Bradyrhizobium *, photosynthesis is conferred by a photosynthesis gene cluster (PGC) of 45–50 kb in size [23, 31]. The cluster encodes all the products needed for successful photosynthesis including bacteriochlorophyll biosynthesis (encoded by the *bch* genes), carotenoid biosynthesis (encoded by the *crt* genes), light-harvesting polypeptides (encoded by the *pufLM* genes) and the reaction centre subunits (encoded by the *pufBA* genes), as well as those involved in regulation (e.g. bacteriophytochrome encoded by *BrBphP* and transcription factors encoded by *ppsR1, ppsR2* and *acsF*) [26, 31]. In terms of gene organization, this PGC generally resembles that of other photosynthetic *Pseudomonadota* (* Proteobacteria *), although regulation of the *Bradyrhizobium* PGC requires specific light conditions to initiate the process, controlled by the bacteriophytochrome photoreceptor (BrBphP).

The *Bradyrhizobium* PGC was first identified in the chromosome of strain BTAi1, which is a member of the Photosynthetic supergroup and a symbiont of *Aeschynomene indica* (Indian jointvetch) [32]. Since then, it has also been identified in several other *Bradyrhizobium* symbionts of *Aeschynomene* (e.g. strains ORS278 and ORS375) [23, 31, 33]. Outside the Photosynthetic supergroup of *Bradyrhizobium *, the PGC has rarely been encountered and its discovery has largely been attributed to whole genome sequencing efforts [20]. Until recently, the non-nodulating strain S23321 (now known as *Bradyrhizobium cosmicum *) was the only member of the so-called ‘* B. japonicum* supergroup’ whose genome was known to contain a PGC [20, 30]. However, with the increased availability of whole genome sequence data, particularly from recently described rhizobial species [34], a version of the PGC has been identified in *B. amphicarpaeae* 39S1MB^T^ [28], *B. betae* PL7HG1^T^ [29] and *B. cosmicum* 58S1^T^ [30]. The PGC is also present in other members of the *Nitrobacteraceae,* including species of the genera *Rhodopseudomonas *, *Tardiphaga* and *Rhodoplanes* [6, 7, 35].

Various authors have highlighted the patchy distribution of the PGC and its genes within and among the *Bradyrhizobium* supergroups [20, 28, 29], but no previous study has utilized a comparative genomics approach for explaining the patterns observed. Therefore, the aim of the current study was to explore the distribution and evolution of the PGC across the species of *Bradyrhizobuim*. To do this, the cluster was identified from all the *Bradyrhizobium* genomes in the Photosynthetic supergroup and other supergroups for which full genome sequences are available. Genomes containing the PGC were compared with regard to genomic location, gene content and synteny of their clusters. Lastly, the evolutionary history of the photosynthetic genes was investigated. The combination of comparative genomics with comprehensive phylogenetic data thus allowed for a well-resolved evolutionary history of this interesting biological trait in *Bradyrhizobium *.

## Methods

### Identification and comparison of PGC-containing regions in *Bradyrhizobium* and related genera

A collection of PGC-containing genome sequences was compiled in CLC Main Workbench v.8.1 (CLC Bio) using data available from the National Center for Biotechnology Information (NCBI; http://www.ncbi.nlm.nih.gov/). This was done by using the known PGCs of *Bradyrhizobium* strain ORS278 and *B. cosmicum* strain S23321 [20, 30, 33] as reference sequences for those encoded by the Photosynthetic and *B. japonicum* supergroups, respectively. From these two clusters, the *bchBCDFGHLNXYPZ* (bacteriochlorophyll biosynthesis), *crtBCDEFI* (carotenoid biosynthesis) and *pufBALM* (light-harvesting complex biosynthesis) genes were used as queries in BLASTn [36] searches against *Bradyrhizobium* sequences in NCBI’s Whole-Genome Shotgun (wgs) database default parameters [i.e. maximum target sequences of 100, expected (E) threshold of 0.05 and maximum matches in a query range]. Another round of similar BLASTn searches was performed, but with search parameters limited to *Nitrobacteraceae* and excluding *Bradyrhizobium *. In both rounds of BLASTn searches, those genomes containing all the query genes were identified and retrieved. Additionally, the full genome sequences for outgroup taxa known to have photosynthesis genes were retrieved for the families *Boseaceae* and *Methylobacteriaceae* (both members of the order *Hyphomicrobiales *), as well as *Paracoccaceae* (order *Rhodobacterales *, class *Alphaproteobacteria *) [37–39].

Within the collection of genomes, the PGC and genes flanking it were identified for analysis. For this, PGC-containing genomic regions were located using the ORS278 PGC in local BLASTn searches with default settings in CLC Main Workbench. Start and end positions of the cluster in *Bradyrhizobium* and other *Nitrobacteraceae *, as well as the outgroup taxa, were determined based on the PGC of *Bradyrhizobium* strain ORS278 and previous studies [20, 38]. The identified PGC, together with the region spanning 15 genes directly up- and downstream of it, was then extracted from the respective genomes for further study. For those genomes in which these regions were not annotated, genes in the relevant regions were first annotated using the RASTtk (Rapid Annotations using Subsystems Technology toolkit) pipeline v.1.073 [40–42] implemented in KBase [43]. In those cases where the PGC and flanking regions were split across contigs, the sequences were manually scaffolded using the PGC of *Bradyrhizobium* strain ORS278 as a reference.

To compare the genomic location, gene content and gene order within the identified PGC and flanking genes among all genomes, a synteny plot was constructed using EasyFig v.2.2.2 [44]. The PGC and flanking regions from *Boseaceae, Methylobacteriaceae* and *Paracoccaceae* were included as outgroups for the analysis [38]. These regions were added to the *Bradyrhizobium* and *Nitrobacteraceae* datasets, and similarity between the genomes were determined using BLASTn analysis executed within EasyFig using a maximum E-value cut-off of 0.001.

### Phylogenetic analysis and ancestral state reconstruction

Two whole genome-based phylogenies were generated. The first served as a species tree for *Bradyrhizobium* and included all available genome sequences for this taxon (as of October 2021), as well as representatives of closely related genera within the *Nitrobacteraceae* (*Nitrobacter, Rhodopseudomonas, Tardiphaga*, *Afipia, Oligotropha, Pseudorhodoplanes, Rhodoplanes, Pseudolabrys, Labrys* and *Variibacter *), *Methylobacteriaceae* (* Methylobacterium *), *Boseaceae* (* Bosea *) and *Paracoccaceae* (* Rhodobacter *). The second genome-based phylogeny included only the PGC-containing *Bradyrhizobium* genomes, other *Nitrobacteraceae* representatives and the outgroup taxa used before. From the genomes of both taxon sets, the Up-to-date Bacterial Core Gene (UBCG) pipeline was used to identify, align and extract the sequences for 92 universal genes [45]. Each nucleotide dataset was concatenated and partitioned using FASconCAT-G v.1.02 [46]. The two concatenated datasets were then subjected to maximum-likelihood (ML) analysis in RAxML v.8.2.1 [47], using the General Time Reversible (GTR) substitution model [48] with independent parameter optimization for each gene partition [49]. For estimating branch support, these ML analyses included non-parametric bootstrapping based on 1000 repetitions [47].

Reconstruction of the ancestral state for the PGC was performed using the MrBayes Ancestral States with R (MBASR) toolkit [50]. For this, the topology of the *Bradyrhizobium* species tree (generated from the concatenated nucleotide dataset) was used as a backbone onto which the character states were mapped. Here, the respective character states were regarded as unordered, discrete, and denoted based on the presence or absence of the PGC. MBASR then utilized 1000 Markov chain Monte Carlo samples (=100 000 generations) and continuous-time Markov modelling [51, 52] across the species tree to provide posterior probability estimates for character states at the nodes.

To investigate the evolution of the PGC in *Bradyrhizobium *, phylogenetic analyses were performed on the *bchIDOCXYZFNBHLMPGEF*, *crtIBCDEF*, *pufBALM*, *ppsR1, ppsR2, BrBphP* and *acsF* genes. For this, the protein sequences of *Bradyrhizobium cosmicum* S23321 and *Bradyrhizobium* strain ORS278 were used to identify homologues in NCBI’s non-redundant protein database by making use of BLASTp [36] searches. The top blast hits for the two rounds of searches (in all cases limited to 500 bacterial hits) were retrieved and combined to generate a single dataset for each gene, after which duplicate sequences were removed using Geneious v.8 [53]. Individual datasets were then aligned in CLC Main Workbench using muscle (Multiple Sequence Comparison by Log-Expectation) [54]. These datasets were subjected to ML phylogenetic analysis using the IQ TREE web server with default settings [55, 56] to reconstruct individual gene trees. To estimate branch support, all ML analyses included ultrafast bootstrapping [57] and the Shimodaira–Hasegawa-like approximate likelihood-ratio test (SH-aLRT) [58].

## Results

### Multiple *Bradyrhizobium* lineages and other *Nitrobacteraceae* encode a putative PGC

BLASTn searches with known gene clusters as query sequences identified 44 bacterial genomes containing a putative PGC (45–55 kb in size) in publicly available genome repositories (Tables 1 and S1, available in the online version of this article). These included 25 *Bradyrhizobium* genomes, each of which encoded genes with high similarity (percentage identity >85 % and E-value=0) to the target photosynthesis genes of either *Bradyrhizobium* strain ORS278 or *B. cosmicum* S23321 (Table 1 and S1). Homologues with a high similarity to the query sequences were also identified in other genera of the *Nitrobacteraceae *, including *Tardiphaga* (two genomes), *Rhodoplane* s (two genomes), *Pseudorhodoplanes* (one genome) and *Rhodopseudomonas* (six genomes) (Table 1). The remaining PGC-containing genomes were those for the outgroup taxa, i.e. *Bosea* (two genomes), *Rhodobacter* (two genomes) and *Methylobacterium* (four genomes).

**Table 1.** Summarized genome information of *Bradyrhizobium* and outgroup strains investigated in this study

Supergroup	No. of genomes encoding PGC	Total no. of genomes in supergroup*	Source	Presence of nodulation genes†	Genome size range (Mb)	Contig range
Photosynthetic	10	11	*Aeschynomene sensitiva, Aeschynomene indica*	1 of 10	7.3–8.3	1–803
* B. japonicum *	10	271	*Glycine max,* paddy field soil, *Arachis hypogaea*, *Beta vulgaris*, *Arabidopsis thaliana,* hospital shower hose biofilm	3 of 10	7.1–8.3	1–222
* B. elkanii *	5	70	*Asterionella formosa* BG1, tissue specimen from human sample, wastewater	0 of 5	7.1–7.7	1–2463
Outgroup	19	38	Microbial fuel cells, freshwater sediment, Arctic glacier, Liard River hot springs, subsurface in Tinto river, activated sludge from sewage treatment plant, freshwater lake, algal phycosphere, rice stem, leaf tissues of rice, rice grains, spore of *Globus iranicum* var. *tenuihypharum*	0 of 19	4.07–6.9	1–1152

See Table S1 for more information on genomes encoding the PGC.

*Total number of genomes in each supergroup and outgroup at the time of study (see Fig. S3).

†Presence of nodulation genes in genomes encoding the PGC.

Among the 44 genomes examined, the identified PGCs were consistently located on the chromosome and not a plasmid (Tables 1 and S1). Twenty-two of the genomes were designated as complete, with the genomic status of the remainder being noted as incomplete in the respective databases (i.e. consisting of as few as 24 contigs in the case of *Bradyrhizobium* strain MOS003 to as many as 2463 contigs in the case of *Bradyrhizobium* strain 17-4). However, the PGC and flanking regions were mostly identified as being located on single contigs (Table S1). In only seven cases (i.e. *Bradyrhizobium* genomes MOS004, ORS375, STM3809 and 17-4, as well as *Pseudorhodoplanes* genome L.E.AP.45, *Rhodoplanes roseus* DSM 5909 and *Methylobacterium symbioticum* SB0023/3), the cluster spanned across two or more contigs, which were merged using the PGC of *Bradyrhizobium* ORS278 as reference.

The PGC-containing *Bradyrhizobium* genomes mostly originated from strains that were isolated from nodules of legumes (15 genomes) or the tissues of non-leguminous plants (five genomes). Two genomes came from clinical strains, while the strains of another three were isolated from wastewater, a diatom and an unknown host, respectively (Tables 1 and S1). Additionally, of the 25 PGC-containing *Bradyrhizobium* genomes, ten came from strains in the Photosynthetic supergroup, ten from the *B. japonicum* supergroup and five from the *B. elkanii* supergroup.

### Two PGC types are found in *Bradyrhizobium* and other *Nitrobacteraceae*

**Fig. 1.**
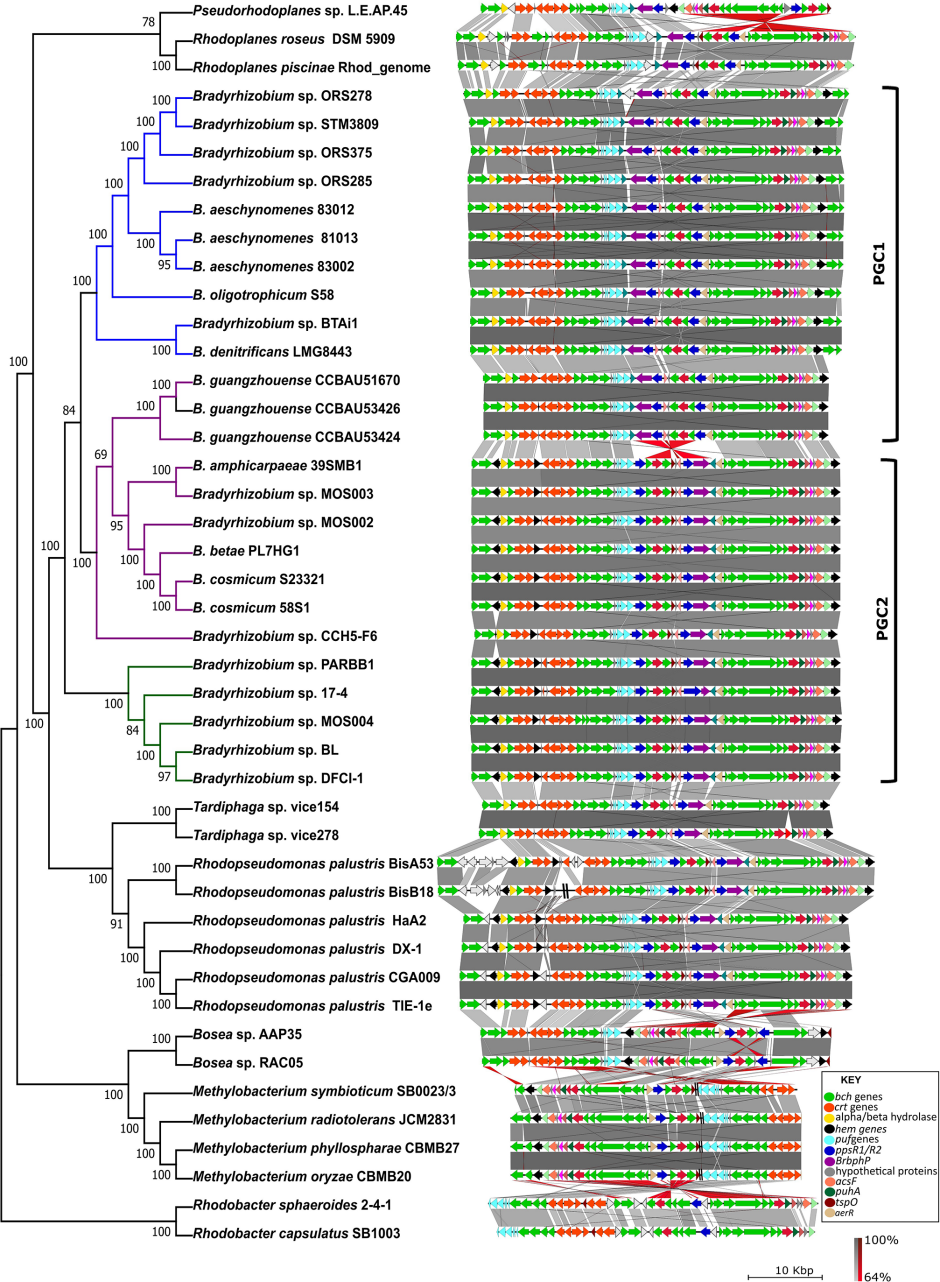
Comparison of the photosynthesis gene clusters (PGCs) identified in *Bradyrhizobium *. Representative strains possessing the PGC from *Nitrobacteraceae* (*Rhodopseudomonas, Tardiphaga, Rhodoplanes* and *Pseudorhodoplanes *), *Methylobacteriaceae* (* Methylobacterium *), *Boseaceae* (* Bosea *) and *Paracoccaceae* (* Rhodobacter *) were included for outgroup purposes. Coloured arrows indicate genes consisting of the PGC and double black lines indicate the presence of additional genes not part of the PGC. Genes are coloured according to biological categories: green, bacteriochlorophyll biosynthesis (*bch*); orange, carotenoid biosynthesis (*crt*); light blue, light-harvesting and photosynthesis reaction centre (*puf*); dark blue, transcriptional regulators (*ppsR*1 and *ppsR*2); grey, hypothetical genes; and white, additional genes possibly not part of the gene cluster. The red region between strains indicates inversion events. Grey regions between the clusters indicate perfect synteny of genes determined by BLASTn. Functions of these genes are presented in Fig. S1 and Table S1.

While all contained the expected *bch*, *crt*, *puf*, *pps* and *BrBphP* genes, numerous differences among the PGC of the respective taxa were observed. These included gene rearrangements within the predicted PGC, with some occurring in *Bradyrhizobium *, others in *Nitrobacteraceae* and yet others in the outgroup genera (Fig. 1 and Table S2). Within *Bradyrhizobium* specifically, genomes harboured one of two alternative forms of the PGC (denoted as PGC1 and PGC2). Overall, the gene organization within PGC1 was conserved and highly syntenic among 13 of the *Bradyrhizobium* genomes including the cluster of reference strain ORS278. Strains encoding this cluster belonged to the Photosynthetic supergroup and the three genomes of *B. guangzhouense* situated in the *B. japonicum* supergroup (Fig. 1). Here, the presence of *bchEJ* genes (encoding Mg-protoporphyrin IX monomethyl ester oxidative cyclase and 3-vinyl bacteriochlorophyllide hydroxylase) in PGC1 of the Photosynthetic supergroup strains distinguished it from the cluster of the *B. guangzhouense* strains (Figs 1 and S1).

The gene content and organization in PGC2 were also conserved and highly syntenic among the other 12 *Bradyrhizobium* genomes, which included *B. cosmicum* S23321 used as the reference. This also included the remaining *B. japonicum* supergroup strains, as well as all those from the *B. elkanii* supergroup (Figs 1 and S1). Overall, PGC2 differed from PGC1 in having additional *hemE* (uroporphyrinogen decarboxylase) and *hemF* (oxygen-dependent coproporphyrinogen) genes nested within the first set of *bch* genes and *crt* genes, respectively (Figs 1 and S1 and Table S2). PGC2 also lacked the *bchEJ* genes occurring in PGC1.

Comparison of PGC1 and PGC2 indicated an inversion between a subset of genes in the two clusters (Figs 1 and S1). The inverted genes included *BrBphP* (encoding the bacteriophytochrome light sensor), *ppsR2* (encoding a transcriptional repressor), *cycA* (encoding cytochrome c), *bchP* (encoding geranylgeranyl reductase), an MFS (major facilitator superfamily) transporter gene, *bchG* (encoding bacteriochlorophyll synthase) and *ppR1* (encoding a transcriptional activator). PGC2 further included an extra gene (*tspO* encoding a tryptophan-rich sensory protein) within the inverted region.

The gene content and arrangement of the cluster for most of the other *Nitrobacteraceae* were syntenic either to PGC1 or to PGC2. For *Tardiphaga* and *Rhodopseudomonas *, the cluster was similar to PGC2, but differed slightly in gene arrangement, with *Tardiphaga* lacking the *hem* genes and *Rhodopseudomonas* containing an insertion in the first set of *bch* genes, immediately upstream of the nested *hem* gene (Fig. 1). For *Rhodoplanes* and *Pseudorhodoplanes *, the cluster was similar to PGC1. However, in *Rhodoplanes* it has additional hypothetical genes and the *bchEJ* genes are nested among the *crt* genes (Fig. 1), while there is an inversion towards the end of the *Pseudorhodoplanes* cluster. Outside of *Nitrobacteraceae,* the PGC architecture mostly followed generic relationships (i.e. similar gene content and organization between strains of the same genus), with the PGC of *Methylobacterium* being interrupted by a gene region of >800 kb. Also, regulatory genes such as *bphP* and *ppsR* were missing from the PGC in these genera (i.e. *Bosea* spp., *Methylobacterium* spp. and *Rhodobacter* spp.) (Figs 1 and S1 and Table S2).

### Genomic neighbourhood of the PGC differs among lineages

**Fig. 2.**
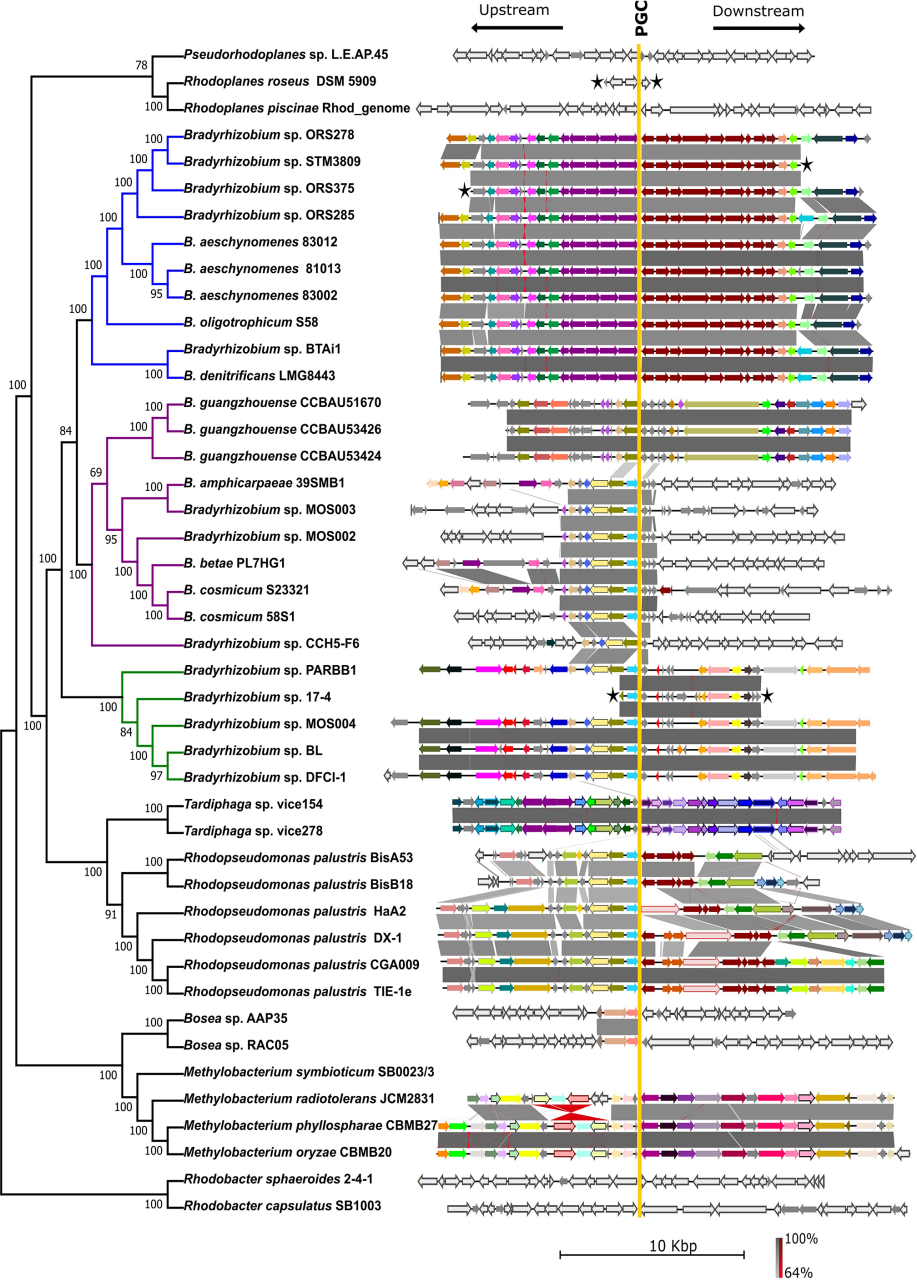
Comparison of the 15 genes immediately flanking the photosynthesis gene cluster (PGC) upstream and downstream identified in *Bradyrhizobium* and representative strains from *Nitrobacteraceae* (*Rhodopseudomonas, Tardiphaga, Rhodoplanes* and *Pseudorhodoplanes *), *Methylobacteriaceae* (* Methylobacterium *), *Boseaceae* (* Bosea *) and *Paracoccaceae* (* Rhodobacter *). Grey regions between the clusters indicate synteny of genes determined by BLASTn. Genes that share homology are all indicated by the same colour. Genes with unique functions (i.e. genes that do not share homology) are indicated in green. Strains with contigs that are too short to allow for the indication of the entire set of 15 genes flanking on either side is indicated with a black star at the breakpoint. The functions of these genes are indicated in Fig. S2 and Table S2.

The content and organization of genes flanking the PGC were generally conserved but not identical within each of the respective *Bradyrhizobium* supergroups (Figs 2 and S2 and Table S3). For the Photosynthetic supergroup, genes involved in histidine utilization (*hutUHIFC*) occupied the upstream flank of PGC1, while genes involved in the Calvin cycle were located downstream of it. The latter genes included those encoding for class 1 fructose-bisphosphatase, phosphoribulokinase, transketolase, fructose-bisphosphate aldolase class II, the large and small subunits of ribulose-1,5-bisphosphate carboxylase-oxygenase (rubisco) and the CbbX ATPase needed for activation of rubisco. Although conserved among the three *B. guangzhouense* (* B. japonicum* supergroup) genomes, their PGC1 flanks differed from those of the Photosynthetic supergroup, with the upstream flanking region mostly containing genes of unknown function and the downstream genes encoding proteins involved in dehydrogenase enzyme family, signalling and stress (Fig. S2 and Table S3).

The PGC2 flanks in the *B. japonicum* supergroup varied substantially, with conserved sequences limited to regions immediately adjacent to the cluster (Figs 2 and S2, and Table S3). By contrast, the flanking regions of PGC2 in the *B. elkanii* supergroup appeared to be highly conserved, with extended regions of synteny both upstream and downstream of it. For the most part, the flanking regions in the *B. japonicum* and *B. elkanii* supergroups included hypothetical genes, genes with unknown functions, and others only annotated to gene family-level (e.g. GNAT family *N*-acetyltransferase, ATP-binding cassette transporters, SDR family oxidoreductase, GNTR family of transcriptional factors, TetR-family transcription factors AAA family ATPase) (see Fig. S2 and Table S3).

Gene organization of the flanking regions for the remainder of *Nitrobacteraceae* were conserved within each genus but varied among genera. Like the *Bradyrhizobium* Photosynthetic supergroup, *Rhodopseudomonas* also contained some of the genes involved in the Calvin cycle (i.e. genes encoding the large and small rubisco subunits and CbbX), despite its PGC architecture being different. In *Rhodoplanes* and *Pseudorhodoplanes* the cluster was flanked by unique sets of genes (Fig. S2 and Table S3). As for the outgroup genera, no gene conservation in the PGC flanking regions of *Bosea* and *Rhodobacter* was observed. However, those flanking the PGC of *Methylobacterium* were syntenic (* M. symbioticum* SB0023/3 was excluded from the comparison due to the incompleteness of the genome assembly). Most of these flanking genes were involved in signalling, transcription regulatory activities, chemotaxis, oxidoreductase and transferase activity, and xenobiotic transport, but many of the genes have unknown functions (Fig. S2 and Table S3).

Mobile elements were also present among the first 15 genes flanking the cluster (Fig. 2). These mobile elements were predominantly seen in the PGC upstream and downstream flanking regions of *Bradyrhizobium* genomes within the *B. elkanii* supergroup. (e.g., see red genes in the upstream and downstream flanking regions Figs 2 and S2; Table S3). All the mobile elements detected belonged to the IS3 family transposase according to the annotated GenBank sequence file of the respective genomes.

### Photosynthesis in *Bradyrhizobium* probably has a complex evolutionary history

**Fig. 3.**
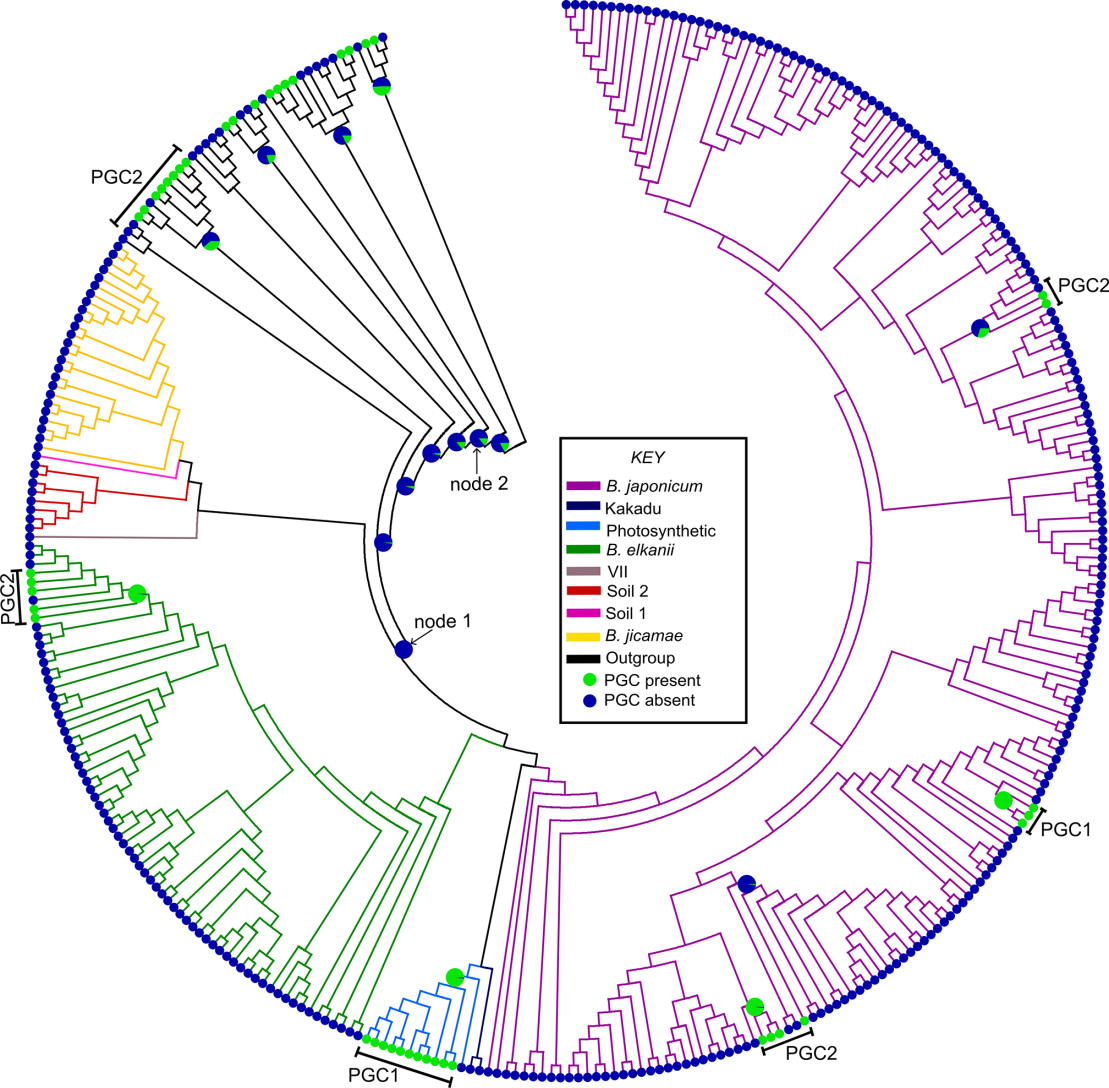
Ancestral state reconstruction of the PGC in *Bradyrhizobium* and outgroup genomes, *Bradyrhizobiaceae */* Nitrobacteraceae* (*Rhodopseudomonas, Tardiphaga, Rhodoplanes* and *Pseudorhodoplanes *), *Methylobacteriaceae* (* Methylobacterium *), *Boseaceae* (* Bosea *) and *Paracoccaceae* (* Rhodobacter *). The PGC character states are mapped onto the *Bradyrhizobium* species phylogeny (Fig. S3) according to the presence or absence of the gene cluster. The key indicates the different lineages, as well as the absence and presence of a PGC (see Fig. S5 and Table S4 for posterior probability values).

Ancestral character state reconstruction analysis with the MBASR toolkit suggested that the PGC was probably acquired horizontally by the different lineages of *Bradyrhizobium* (Fig. 3). Bayesian modelling against the *Bradyrhizobium* species tree (inferred from the concatenated 92 UBGC genes) indicated that the cluster was absent [posterior probability (PP)=1.00] from the ancestor of the genus (node 1 in Fig. 3). Within *Bradyrhizobium *, single acquisition events in the ancestor of the Photosynthetic supergroup (PP=0.98) and in the ancestor of the *B. guangzhouense* lineage (PP=0.99) explain the presence of PGC1 in these taxa (Figs 3 and S5, and Table S4). Also, the presence of PGC2 in the *B. elkanii* supergroup was probably due to an acquisition in only one of its lineages (PP=0.99) (Figs 3 and S5, and Table S4). This was also true for the remaining instances, where PGC2 was acquired only by specific lineages within the *B. japonicum* supergroup (Fig. 3). Whether the ancestor of *Nitrobacteraceae* (node 2) contained the PGC is uncertain (PP=0.14) and would need a broader and more representative taxon sampling across the family (Figs 3 and S5, and Table S4).

**Fig. 4.**
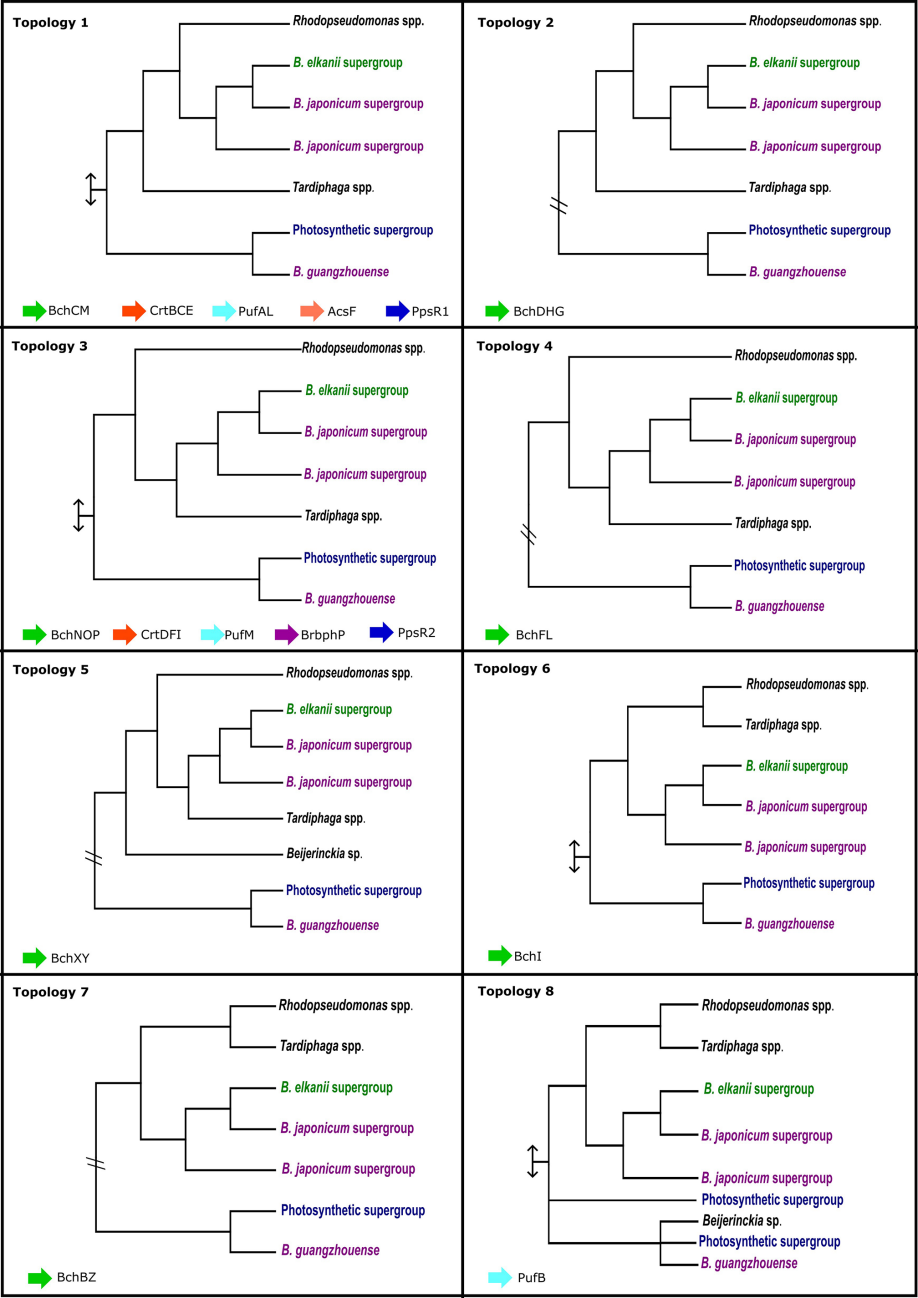
Eight summary trees representing the phylogenies of 29 proteins involved in photosynthesis (see Figs 4.1–4.29). Individual gene trees were inferred by combining ML phylogenetic analysis of datasets consisting of various photosynthesis gene-containing bacterial taxa as well as sequences identified using BLASTp searches against NCBI’s non-redundant database. The eight patterns shown are representatives of common branching patterns seen of strains in PGC1 and PGC2 and their closest relatives *Rhodopseudomonas* and *Tardiphaga *. The proteins sharing a similar topology are indicated at the bottom of each box (i.e. topology 1 is shared by proteins BchCM, CrtBCE, PufAL, AcsF and PpsR1, topology 2 is shared by BchDHG, topology 3 is shared by BchNOP, CrtDFI, PufM, BrbphP and PpsR2, topology 4 is shared by BchFL, topology 5 is shared by BchXY, topology 6 is represented by BchI, topology 7 is shared by proteins BchBZ and topology 8 is represented by PufB protein. The symbol < – > indicates that the summary tree is part of a larger phylogenetic tree and // represents a break between strains of PGC1 and PCG2 indicating that there are additional taxa between the groups.

To further explore the evolutionary history of the *Bradyrhizobium* PGC, ML-based phylogenies were inferred for the individual genes (Figs S4.1–S4.29) and then compared to the species tree (Fig. S3). However, to facilitate comparisons between genes, relevant taxon relationships were extracted from these large phylogenies. For the 29 genes included in PGC1 and PGC2, this resulted in eight distinct topologies, two of which were more frequently recovered (i.e. nine genes for Topology 1 and nine genes for Topology 3 in Fig. 4). In all topologies, except the one observed for *pufB*, the taxa containing PGC1 (i.e. the Photosynthetic supergroup and *B. guangzhouense *) formed a distinct and monophyletic group. This could be indicative of horizontal acquisition in the ancestor of the Photosynthetic lineage, and a subsequent horizontal transfer to *B. guangzhouense *. Also, the sequences from PGC1 frequently (15 out of 29 sequences) grouped closely with those from *Beijerinckiaceae* (order *Hyphomicrobiales *) or *Rhodospirillales* (class *Alphaproteobacteria *) (Figs S4.1, S4.4, S4.7, S4.9, S4.11–S4.12, S4.14–S4.15, S4.17–S4.18, S4.20, S4.23–S4.26).

The PGC2-containing taxa were recovered as monophyletic in all 29 phylogenies (Figs S4.1–S4.29). Sequences from the *B. elkanii* supergroup were consistently nested within the *B. japonicum* supergroup (Figs S4.1–S4.6, S4.8, S4.11, S4.13–4.19, S4.23–S4.24, S4.26–S4.27, S4.29) with ultrafast bootstrap and SH-aLRT support values of >90 %. This grouping pattern is incongruent with the *Bradyrhizobium* species tree (Fig. S3) where the *B. japonicum* and *B. elkanii* supergroups represent separate lineages. This is consistent with the PGC2 variant being horizontally transferred from a member of the *B. japonicum* supergroup to an ancestor of the PGC-containing lineage in the *B. elkanii* supergroup. In some instances the *Tardiphaga* genomes also grouped closely to *B. elkanii* and *B. japonicum* (i.e. topologies 3, 4 and 5), and in other instances *Rhodopseudomonas* grouped sister to the *B. elkanii* and *B. japonicum* lineages (i.e. topologies 1 and 2), with only four phylogenies (i.e. *bchI*, *bchB*, *bchZ* and *pufB*, represented by topologies 6, 7 and 8) showing the expected sister group relationship between *Tardiphaga* and *Rhodopseudomonas* (see Fig. S3). This again suggested horizontal gene transfer among genomes of these three genera. Furthermore, in two phylogenies, all *Bradyrhizobium* sequences grouped together, obscuring clear phylogenetic signals, supporting the possibility of horizontal gene transfer of PGC among *Bradyrhizobium *, *Tardiphaga* and *Rhodopseudomonas* and of lateral exchange of particular photosynthesis genes among members of *Bradyrhizobium* (see Figs S4.27, S4.29). Note that these groupings could have been impacted in some cases by phylogenetic artefacts involving character sampling bias, stochastic error or incomplete lineage sorting.

## Discussion

This study showed that 25 publicly available *Bradyrhizobium* genomes, spanning multiple lineages, encode one of two apparently complete versions of the PGC. One of these, PGC1, showed high levels of synteny between the Photosynthetic supergroup and the genomes of three strains of *B. guangzhouense* in the *B. japonicum* supergroup. The other version, PGC2, was highly syntenic among the remainder of the genomes from the *B. japonicum* supergroup and from the *B. elkanii* supergroup. Overall, these findings are thus consistent with those of previous studies on the genomes, respectively, of the nodulating strains (ORS278 and BTAi1) in the Photosynthetic supergroup and the non-nodulating *B. cosmicum* S23321 [20, 23, 30, 31]. Also, as shown before [20], the major differences observed between the two PGCs involved inversions between *BrBphP* and *ppR1* and lack of *hem* genes (excluding *hemA*) in PGC1, with the lack of *bchEJ* in PGC2 (see Fig. 1).

Outside of *Bradyrhizobium *, the other *Nitrobacteraceae* genomes examined also encoded one of two forms of the PGC. Based on gene content, organization and phylogeny, the cluster found in the *Rhodoplanes* and *Pseudorhodoplanes* genomes were most similar to PCG1 of *Bradyrhizobium *. Those identified in the *Rhodopseudomonas* and *Tardiphaga* genomes were most similar to PGC2. Among *Nitrobacteraceae *, however, our knowledge of photosynthesis as a trait is limited to strains of *Rhodoplanes *, *Rhodopseudomonas* and Photosynthetic supergroup *Bradyrhizobium *. Their photosynthesis genes, photosynthesis activity and photopigments have been studied [7, 35, 59], while the PGC’s functionality in *Tardiphaga* and *Pseudorhodoplanes* needs verification [6].

Based on previous experimental work, the photosystem encoded by *Bradyrhizobium *’s PGC2 (should it be active) is expected to be regulated markedly differently from the one encoded by PGC1. For example, it was shown that the PGC1 system (found in the *Bradyrhizobium* Photosynthetic supergroup) is expressed under aerobic light conditions, while the system in *Rhodopseudomonas palustris* (i.e. encoded by a gene cluster with high similarity to PGC2 of *Bradyrhizobium *) is expressed under anaerobic light conditions [31, 60]. In both systems the expression of the photosystem is mediated by the light-sensing ability of the bacteriophytochrome photoreceptor (BphP) and two transcriptional regulators PpsR1 and PpsR2. In the PGC1 system, these two regulators exert antagonistic effects, with PpsR2 being a repressor and PpsR1 being an activator of photosynthesis gene expression [26, 61]. In *Rhodopseudomonas palustris *, however, both PpsR1 and PpsR2 apparently repress transcription and for photosynthesis gene expression to occur, BphP needs to interact with either or both of the repressors [62]. Future studies should therefore seek to determine whether the PGC2 photosystem of *Bradyrhizobium* and the PGC2-like clusters of *Tardiphaga* are also regulated via the bacteriophytochrome and dual transcriptional factors as in the case of *R. palustris *.

*Bradyrhizobium* strains encoding PGC1 all represent nodulating symbionts of legumes in the tribe Dalbergieae [23, 24, 32, 63–66]. Those strains belonging to the Photosynthetic supergroup were isolated from root or stem nodules of *Aeschynomene* and the PGC1-containing *B. guangzhouense* strains came from *Arachis hypogaea* root nodules. Strains from the Photosynthetic supergroup utilize a Nod-factor-dependent or Nod-factor-independent process to infect the plant roots via the so-called ‘crack entry’ mechanism (i.e. intercellular infection, allowing direct access to the host’s cortical cells), rather than through a Nod-factor-dependent process involving infection threads of root hairs as is the case for other rhizobia [67–69]. In the bacteria belonging to the Photosynthetic supergroup, it has been speculated that energy gained from photosynthesis is used to stimulate and support nitrogen fixation [31]. Likewise, *Bradyrhizobium* strains associated with *Arachis hypogaea* are also known to infect the plant via crack entry [69], but whether the PGC1-containing *B. guangzhouense* strains photosynthesize *in planta* and if photosynthesis also has a stimulating role on nitrogen fixation remain to be determined. Similar uncertainties also remain regarding the role of *bchEJ* in bacteriochlorophyll biosynthesis, as these genes are present in the *Aeschynomene* symbionts but absent from the PGC1-containig *B. guangzhouene* strains and many other phototrophic *Alphaproteobacteria* not included here [38].

PGC2-containing genomes from the *B. japonicum* and *B. elkanii* supergroups were isolated from various ecological niches. In the case of the *B. japonicum* supergroup, these ranged from roots or root nodules of legumes or non-legumes [20, 28–30] to the biofilm inside a hospital shower hose (NCBI: txid1768753). In the *B. elkanii* supergroup, niches included a sewage treatment plant [70], river sediment [71], human biospecimen associated with cord colitis syndrome [72], a culture of the diatom *Asterionelle formasa* and *Arabidopsis* roots [73]. These *Bradyrhizobium* strains might have acquired the PGC from other members within their respective environments, which is not uncommon for some of these ecological niches associated with aquatic sediments or biofilms [59, 74]. Interestingly, the PGC2-containing strains mostly also lack *nod* genes, while some additionally lack genes needed for nitrogen fixation. Although it is not clear why these strains contain a PGC and if it is functional, these results highlights how our knowledge of *Bradyrhizobium* is mainly driven by and limited to its symbiosis ability with legumes [21, 73].

Ancestral character state reconstruction and phylogenetic analyses of all PGC coding sequences showed that the evolutionary origin of photosynthesis in *Bradyrhizobium* entails multiple horizontal acquisitions coupled with vertical inheritance. Whether the ancestral *Nitrobacteraceae* lineage encoded a PGC is uncertain, as multiple subsequent losses in most genera would have needed to occur to explain its distribution among extant taxa. However, photosynthesis genes and clusters encoded by numerous *Pseudomonadota* (* Proteobacteria *) commonly experience horizontal gene transfer [6, 38, 75, 76]. Therefore, multiple horizontal acquisitions of the PGC across *Bradyrhizobium* and the broader *Nitrobacteraceae* represent a more plausible explanation of the distribution patterns observed. This is evidenced by the phylogenies for individual photosynthesis genes, the PGC’s architecture in *Bradyrhizobium* and close relatives, and the presence of mobile elements in the PGC’s flanking regions (see Figs 1, 2 and 4). Further refinement of the evolutionary hypotheses underpinning photosynthesis and the occurrence of a PGC in *Nitrobacteraceae* would require exploration of genome data for more members of the family, especially for taxa outside *Bradyrhizobium *.

Our results indicate that the ancestor of *Bradyrhizobium* was probably not capable of photosynthesis. This is contrary to previous suggestions that its ancestor was a free-living, photosynthetic bacterium that adapted to the symbiotic lifestyle by acquiring nodulation genes and losing the photosynthesis trait [23, 26, 64]. Various authors have argued that this hypothesis was supported by the fact that photosynthetic strain S23321 of *B. cosmicum* [20] lacked nodulation genes [20], and the occurrence of photosynthetic strains in stem nodules where light is accessible, as well as the ability of some photosynthetic strains to engage in the nitrogen-fixing symbiosis in a Nod-factor-independent manner [23, 26, 64]. Accordingly, selection would have favoured the loss of photosynthesis genes in bacteria where they are no longer useful [23], leading to the PGC’s patchy distribution among extant bradyrhizobia. However, our Bayesian ancestral state reconstruction unambiguously showed that the *Bradyrhizobium* ancestor did not harbour a PGC, but that it was acquired through multiple independent horizontal gene transfer events, a scenario that also provides plausible explanations for the emergence of *Bradyrhizobium *’s two PGC variants.

To conclude, this study is the first to investigate the distribution, genomic location, gene organization, synteny and evolution of the PGC in *Bradyrhizobium *. Analysis of the available genome resources allowed for the evolutionary intricacies underpinning photosynthesis in *Bradyrhizobium* to be unravelled. The work presented can thus serve as a basis to explore details of how the two PGC versions came into existence and whether it occurred in a stepwise process. The latter is important, as the current study only utilized genomes containing the complete gene cluster. In addition, by viewing our findings against a background of previous photosynthesis studies, experimentally tractable models/frameworks might emerge for further exploration of the biological role(s) of PGCs in *Bradyrhizobium* and the *Nitrobacteraceae *.
